# Characteristics and prognosis of EGFR mutations in small cell lung cancer patients in the NGS era

**DOI:** 10.1007/s12094-023-03263-w

**Published:** 2023-07-12

**Authors:** Xiaohong Xie, Guihuan Qiu, Ziyao Chen, Ting Liu, Yilin Yang, Zhixuan You, Chen Zeng, Xinqing Lin, Zhanhong Xie, Yinyin Qin, Yansheng Wang, Xiaodong Ma, Chengzhi Zhou, Ming Liu

**Affiliations:** 1 The First Affiliated Hospital of Guangzhou Medical University, National Center for Respiratory Medicine, National Clinical Research Center for Respiratory Disease, State Key Laboratory of Respiratory Disease, Guangzhou Institute of Respiratory Health, 151 Yanjiang Road, Guangzhou, 510120 China; 2 Nanshan School, Guangzhou Medical University, Guangzhou, China; 3 Key Laboratory of Brain, Cognition and Education Sciences, Ministry of Education, Institute for Brain Research and Rehabilitation, South China Normal University, China Guangzhou,

**Keywords:** Small cell lung cancer, EGFR mutations, EGFR-TKI, Prognosis

## Abstract

**Purpose:**

Targeted therapy has not been effective for small cell lung cancer (SCLC) patients. Although some studies have reported on EGFR mutations in SCLC, a systematic investigation into the clinical, immunohistochemical, and molecular characteristics and prognosis of EGFR-mutated SCLCs is lacking.

**Methods:**

Fifty-seven SCLC patients underwent next-generation sequencing technology, with 11 in having EGFR mutations (group A) and 46 without (group B). Immunohistochemistry markers were assessed, and the clinical features and first-line treatment outcomes of both groups were analyzed.

**Results:**

Group A consisted primarily of non-smokers (63.6%), females (54.5%), and peripheral-type tumors (54.5%), while group B mainly comprised heavy smokers (71.7%), males (84.8%), and central-type tumors (67.4%). Both groups showed similar immunohistochemistry results and had RB1 and TP53 mutations. When treated with tyrosine kinase inhibitors (TKIs) plus chemotherapy, group A had a higher treatment response rate with overall response and disease control rates of 80% and 100%, respectively, compared to 57.1% and 100% in group B. Group A also had a significantly longer median progression-free survival (8.20 months, 95% CI 6.91–9.49 months) than group B (2.97 months, 95% CI 2.79–3.15), with a significant difference (*P* = 0.043). Additionally, the median overall survival was significantly longer in group A (16.70 months, 95% CI 1.20–32.21) than in group B (7.37 months, 95% CI 3.85–10.89) (*P* = 0.016).

**Conclusion:**

EGFR-mutated SCLCs occurred more frequently in non-smoking females and were linked to prolonged survival, implying a positive prognostic impact. These SCLCs shared immunohistochemical similarities with conventional SCLCs, and both types had prevalent RB1 and TP53 mutations.

## Introduction

Small cell lung cancer (SCLC) is a lethal neuroendocrine malignancy, accounts for about 10%-15% of lung cancer. SCLC is characterized by its tendency to metastasize in the early stage, rapidly cell proliferation, and poor prognosis [1]. In the past 30 years, the standard first-line treatment of etoposide plus platinum (EP)for SCLC has not changed. Despite conspicuous response to EP therapy, SCLC relapses 3 months or later and resistance to the initial therapy and most of the patients die to account for their disease ultimately [2]. Last decade, targeted therapies, such as epidermal growth factor receptor tyrosine kinase inhibitors (EGFR-TKIs) have made giant progress in lung cancer. EGFR mutations are commonly detected in most Asian lung adenocarcinoma non-smokers [3] and are closely associated with dramatically response to the EGFR-TKIs [4]. A comprehensive analysis has reported that non-adenocarcinoma lung cancer with EGFR gene mutations less than 5% of all lung cancers [5]. Most recently, gene test performed on 98 surgical sample found 11 EGFR mutations but all presented in combined SCLC [6]. Until now, seldom studies from Taiwan, Japan, China and Italy reported 1.8%-9.1% EGFR mutations [7–11].

No targetable oncogene has been identified in SCLC at present, dozens of clinical trials, which did not select patients based on specific genetic changes, have failed or were abandoned halfway attributed to mini or no impact on progression-free survival (PFS) or overall survival (OS) in SCLC [12]. Current therapies of the SCLC harbored EGFR mutations without standard consensus due to the rare incidence. In addition, current literature data are controversial on the effectiveness of EGFR-TKI in EGFR-mutant SCLC. Up to now, there is no research to explore the efficacy of TKIs combined with EP as the treatment strategy in SCLC patients with EGFR mutations.

Here we reviewed back 57 SCLC patients who were performed next-generation sequencing technology (NGS) in our center. For the first time, we systematically analyzed the clinical, immunohistochemistry and molecular characteristics of SCLC harboring EGFR mutations and the prognosis in such patients. We also systematically evaluated the therapeutic effect of TKIs complied with EP in the first-line treatment of SCLCs with EGFR mutations.

## Materials and methods

### Patients

Total 57 eligible devo SCLC patients who have been conducted on NGS for molecular genetic analysis at the First Affiliated Hospital of Guangzhou Medical University (Guangzhou, China) between September 2016 and September 2021 were enrolled in this study. All patients had de novo diagnosis of SCLC to exclude histological transformation types. Among these patients, 11 SCLCs with EGFR mutations were divided into group A, while 46 patients without EGFR mutation were divided as group B. This series included 54 specimens from biopsy, and 3 from surgically resected tumors and all samples were detected by NGS at the initial biopsy, surgical specimens, pleural effusion or plasma. Histologic diagnosis of SCLC was based on the standard criteria defined by the WHO classification 2015 version [13]. All procedures performed in this study involving human participants were in accordance with the Declaration of Helsinki (as revised in 2013). This study was approved by the Ethics Committee of the First Affiliated Hospital of Guangzhou Medical University.

### Data collection and outcome assessment

The following information was retrospectively collected from the medical records of the patients: patient demographics as sex, age, smoking history, disease stage, anatomy types, histological types, treatments with EGFR-TKIs or systemic therapies, Eastern Cooperative Oncology Group performance status, EGFR mutation type, tumor imaging, tumor response to therapies. The Eastern Cooperative Oncology Group performance status was evaluated prior to treatment strategies. Tumor response was assessed in accordance with the Response Evaluation Criteria in Solid Tumors (RECIST version 1.1) [14]. The objective response rate (ORR) included the percentage of patients who exhibited response (complete or partial). The disease control rate (DCR) corresponds to all cases with partial response (PR), and stable disease (SD). Progression-free survival (PFS) was defined as the time from the initiation of therapy to disease progression or death. Overall survival (OS) was defined as the time from initial therapy to death and last follow-up was March 3, 2022.

### Immunohistochemistry (IHC) staining

**Fig. 1:**
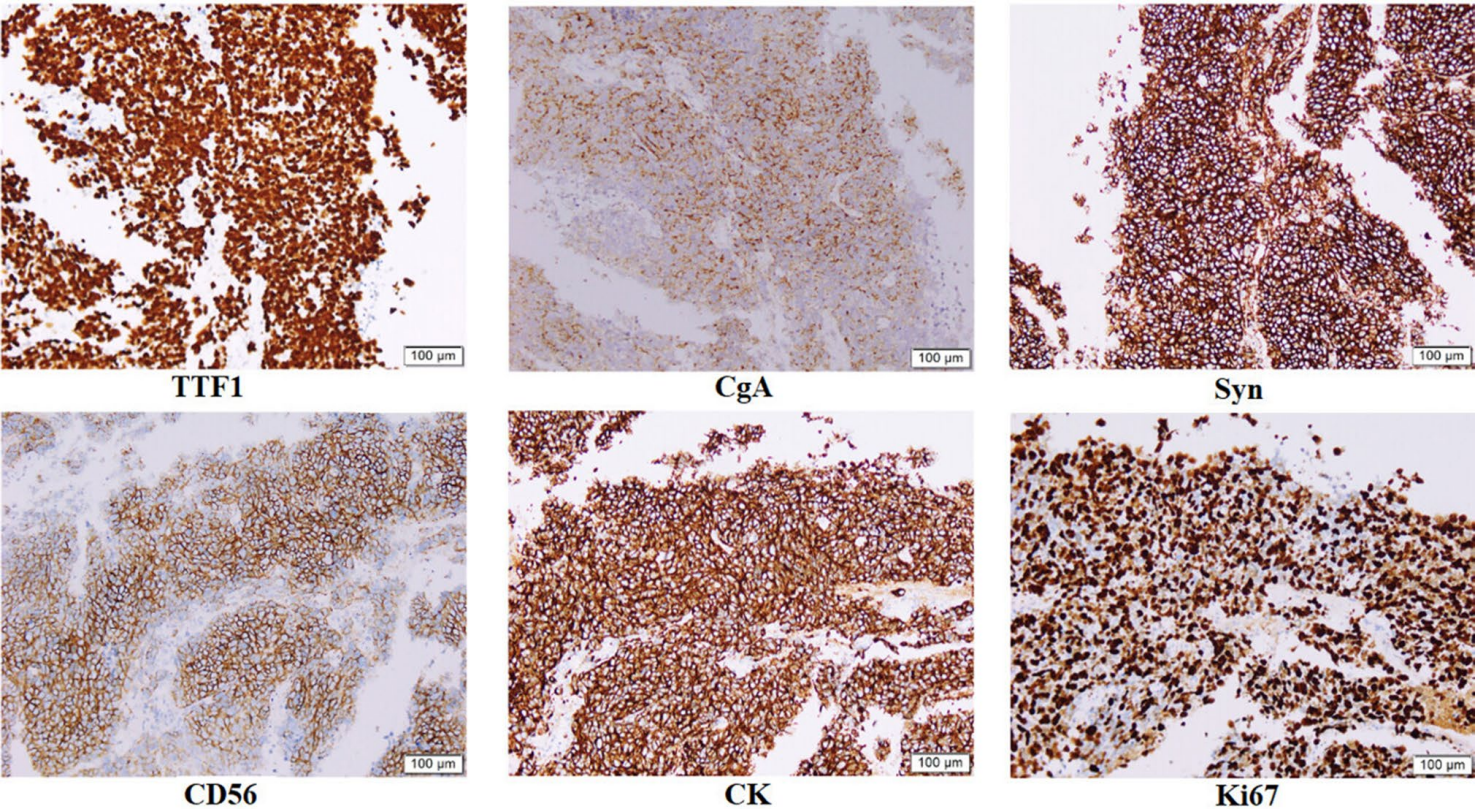
The representative images of these 6 immunohistochemical markers. TTF1, CgA, Syn, CD56, CK, Ki67. Magnification, × 100

Immunohistochemistry stains were conducted on an automated immunostainer machine using the following antibodies: thyroid transcription factor-1 (TTF1) (clone 8G7G3/1), chromogranin A (CgA) (LBP2-Ki67), nerve cell-associated adhesion molecule (CD56/NCAM) (clone MX039), cytokeratin (CK) (clone AE1/AE3), synaptophysin (Syn) (polyclonal RAB-0155) according to the manufacturer’s instructions. For diagnostic purposes on crushed biopsies, Ki67 (clone LBP2-Ki67) was performed in selected cases. Negative and positive controls were included in each batch. For each antibody, the percentage of positive cells and the intensity of staining (0: negative; 1 + : weak; 2 + : moderate; 3 + : strong) were recorded. A tumor was considered positive when at least 10% of the neoplastic cells reacted with an intensity of 2 + or greater on the relevant subcellular localization. The expression of TTF1, CgA, Syn, CD56, CK, Ki67 on cancer samples were scored by two pathologists independently. The representative images of these 6 immunohistochemical markers are shown in Fig. 1.

### Next-generation sequencing (NGS) technology

Capture-based targeted sequencing was performed by Burning Rock Biotech, Guangzhou.

### DNA isolation and capture-based targeted DNA sequencing

DNA isolation and targeted sequencing were performed in Burning Rock Biotech, a commercial clinical laboratory accredited by the College of American Pathologist (CAP) and certified by the Clinical Laboratory Improvement Amendments (CLIA), according to optimized protocols as described previously [15, 16]. Briefly, tissue DNA was extracted from formalin-fixed, paraffin-embedded tumor tissues using QIAamp DNA formalin-fixed paraffin-embedded tissue kit (Qiagen, Hilden, Germany) and circulating cell-free DNA (cfDNA) was extracted from 4–5 ml of plasma samples using a QIAamp Circulating Nucleic Acid kit, according to the manufacturer’s standard protocol (Qiagen, Hilden, Germany). Fragments between 200 and 400 bp from the sheared tissue DNA and cfDNA were purified (Agencourt AMPure XP Kit, Beckman Coulter, CA, USA), hybridized with capture probes baits, selected with magnetic beads, and amplified. Target capture was performed using a commercial panel consisting of 520 genes (OncoScreen Plus), spanning 1.64 megabases of the human genome. The quality and the size of the fragments were assessed by high sensitivity DNA kit using Bioanalyzer 2100 (Agilent Technologies, CA, USA). Indexed samples were sequenced on Nextseq 500 (Illumina, Inc., CA, USA) with paired-end reads and an average sequencing depth of 1,000 × for tissue samples and 10,000 × for liquid biopsy samples.

### Sequence data analysis

Sequence data were mapped to the reference human genome (hg19) using Burrows-Wheeler Aligner version 0.7.10 [17]. Local alignment optimization, duplication marking and variant calling were performed using Genome Analysis Tool Kit version 3.2 (4), and VarScan version 2.4.3 [18]. Tissue samples were compared against their own white blood cell control to identify somatic variants. Variants were filtered using the VarScan fpfilter pipeline, loci with depth less than 100 were filtered out. Base calling in plasma and tissue samples required at least 8 supporting reads for single nucleotide variations and 2 and 5 supporting reads for insertion-deletion variations, respectively. Variants with population frequency over 0.1% in the ExAC, 1000 Genomes, dbSNP or ESP6500SI-V2 databases were grouped as single nucleotide polymorphisms and excluded from further analysis. Remaining variants were annotated with ANNOVAR (2016–02-01 release) [19] and SnpEff version 3.6 [20]. Analysis of DNA translocation was performed using Factera version 1.4.3 [21]. Copy number variations (CNVs) were analyzed based on the depth of coverage data of capture intervals. Coverage data were corrected against sequencing bias resulting from GC content and probe design. The average coverage of all captured regions was used to normalize the coverage of different samples to comparable scales. Copy number was calculated based on the ratio between the depth of coverage in tumor samples and average coverage of an adequate number (*n* > 50) of samples without copy number variations as references per capture interval. CNV is called if the coverage data of the gene region was quantitatively and statistically significant from its reference control. The limit of detection for CNVs is 1.5 for copy number deletion and 2.64 for copy number amplifications.

### Statistical analysis

Continuous variables were analyzed as the median and interquartile range (IQR) or mean and standard deviation. Categorical data were calculated as the frequency (percentage). Either an independent-samples *t*-test or the Mann–Whitney *U* test were used in differences in continuous variables. Correlations between categorical variables were calculated using chi-square and Fisher's exact tests. The ORR and DCR with 95% confidence intervals (CIs) used the binomial exact method. Kaplan–Meier method was used to evaluate OS, PFS, with 95% confidence intervals (CIs), and the log-rank test was used to determine the significance of differences between two subgroups in patients. IBM SPSS Statistics (Armonk, NY), version 25 was used in statistical analyses. A *P* value < 0.05 was considered significant, while all tests were two sides.

## Results

### Patients

Total of 57 patients diagnosed SCLC at our institution were included eligible for this respective study. Among them, EGFR mutations were detected in 11 SCLCs as group A and 46 patients without EGFR mutation as group B. The baseline characteristics were similar between group A and B Table1. However, the distributions of sex, smoking history, disease stage, tumor maximum diameter were significantly different. In the EGFR mutations group, the median age was 62.0 years (range: 33–77 years), six patients (54.5%) were female and 7 patients (63.6%) were non-smoker, 10 patients (90.9%) were extensive stage, while the median age was 65.5 years (range: 50–80 years), 39 patients (84.8%) were male, 33 patients (71.7%) was smokers and 24 patients (52.2%) were limited stage in control group. In addition, the mean of tumor maximum diameter in the EGFR mutation group was larger than the control group. Among patients with group A, EP in 4, EP combined with TKI in 5, EP combined with PD-1inhitbtor in 1 and TKI in 1 as their first-line treatment. In group B, EP only in 14 patients, concurrent chemoradiotherapy in 10, EP + PD-1inhitbtor/PD-L1 inhibitor in 15 as the first line therapy and 7 treatment-naive patients.

**Table 1:** Patient baseline characteristics

Characteristics	Group A	Group B	*P*
Number of patients (*N* = 57)	11	46	
Age (median, range)	62.0 (33–77)	65.5 (50–80)	0.094
Sex (*n*, %)			0.011*
Male	5 (45.5)	39 (84.8)	
Female	6 (54.5)	7 (15.2)	
Smoking history (*n*, %)			0.038*
Yes	4 (36.4)	33 (71.7)	
No	7 (63.6)	13 (28.3)	
ECOG PS (*n*, %)			0.439
0	6 (54.5)	32 (69.6)	
1	5 (45.5)	13 (28.1)	
2	0	1 (2.2)	
Disease stage (*n*, %)			0.016*
LD	1 (9.1)	24 (52.2)	
ED	10 (90.9)	22 (47.8)	
Anatomy type (*n*, %)			0.491
Central	6 (54.5)	31 (67.4)	
Peripheral	5 (45.5)	15 (32.6)	
Tumor max diameter (cm)	6.4 (2.4–11.8)	4.7 (0.9–11.0)	0.013*
Histological type (*n*, %)			0.352
Pure SCLC	10 (90.9)	45 (97.8)	
Combined SCLC	1 (9.1)	1 (2.2)	

*LD* limited disease, *ED* extensive disease

*P < 0.05

### Immunohistochemistry and molecular analysis

The heterogeneity of SCLCs is substantial, and they are not simply a result of combining multiple subtypes or the presence of different mutant molecules. Therefore, we examined whether there is a discernible histological inclination between EGFR-mutated SCLC and typical SCLC using IHC. Results of the IHC analysis are shown in Table 2. Total 57 samples were immunostained for CK, CgA, Syn, TTF1, CD56, Ki67. CK, CgA, Syn, TTF-1, CD56 expressed in 8(72.7%), 7(63.6%), 11(100%), 9(81.8%) and 10(90.9%) respectively in EGFR mutations group. CK, CgA, Syn, TTF-1, CD56 expressed in 43(95.6%), 38(84.4%), 42(93.3%), 40(88.9%), 44(97.8%), respectively in control group. There was no significant difference between wild-type and EGFR mutant SCLC, which means EGFR mutant type was consistent with typical SCLC in IHC features.

**Table 2:** IHC characteristics in patients with EGFR mutations and in control groups

Characteristics	Group A	Group B	*P*
Number of patients	56		
CK, *n* (%)	11	45	0.047*
Positive	8 (72.7%)	43 (95.6%)	
Negative	3 (27.3%)	2 (4.4%)	
CgA, *n* (%)			0.198
Positive	7 (63.6%)	38 (84.4%)	
Negative	4 (36.4%)	7 (15.6%)	
Syn, *n* (%)			1.000
Positive	11 (100%)	42 (93.3%)	
Negative	0	3 (6.7%)	
TTF1, *n* (%)			0.614
Positive	9 (81.8%)	40 (88.9%)	
Negative	2 (18.2%)	5 (11.1%)	
CD56, *n* (%)			0.357
Positive	10 (90.9%)	44 (97.8%)	
Negative	1 (9.1%)	1 (2.2%)	
Ki-67, median	90 (80–95)	80 (50–95)	0.105

*P < 0.05

**Fig. 2:**
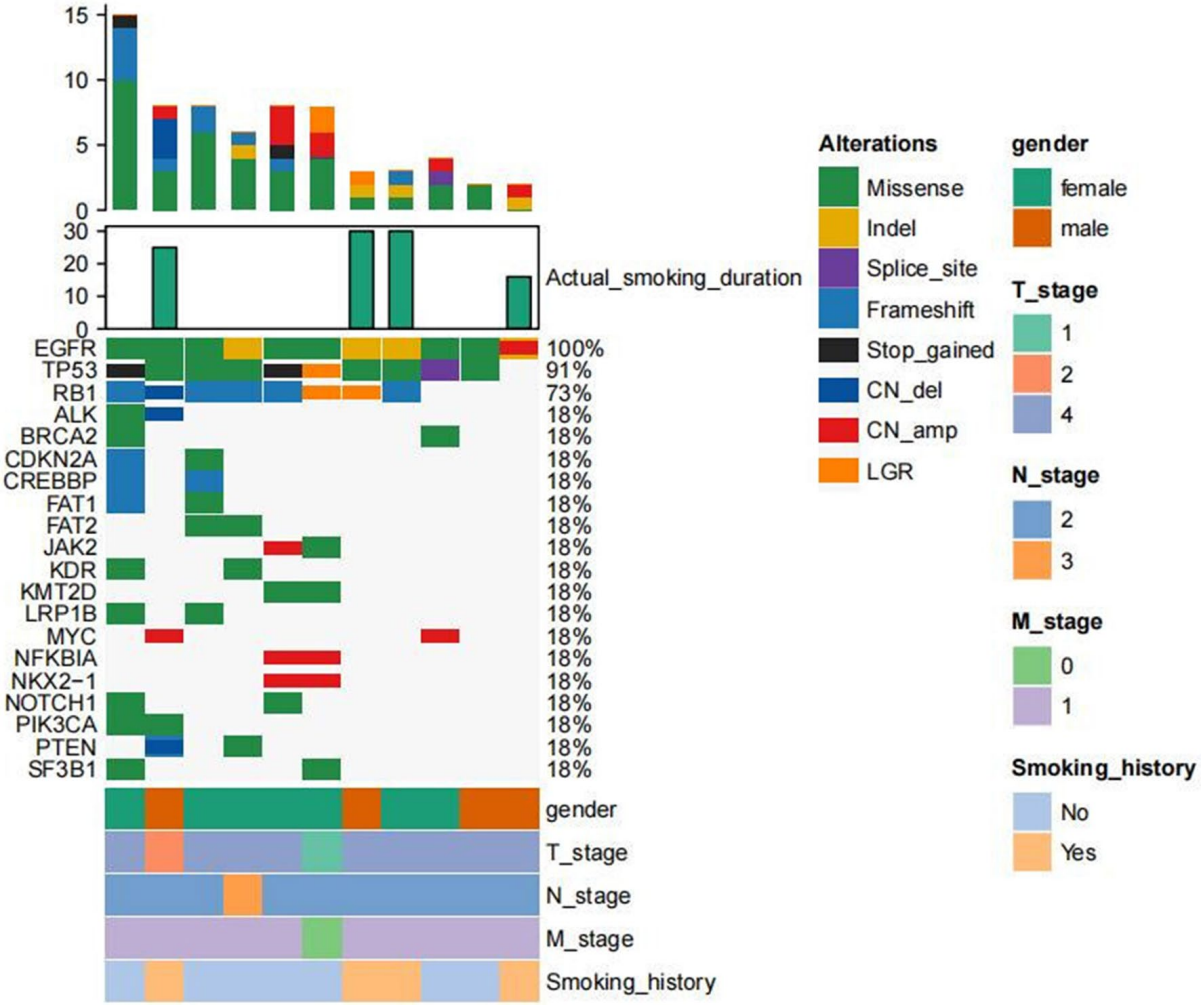
Genomic profiling of 11 patients with EGFR mutations in group A, each column was the relevant tumor samples of the 11 patients, the mutations detected for a given gene was exhibited on each row, respectively, and the proportions of them was shown on the right axis. Green indicated missense, yellow indicated inframe insertion or deletion, purple indicated splice site, light blue indicated frameshift, black indicated stop gain, dark blue indicated copy number deletion, red indicated copy number amplification, orange indicated large genomic rearrangement

**Fig. 3:**
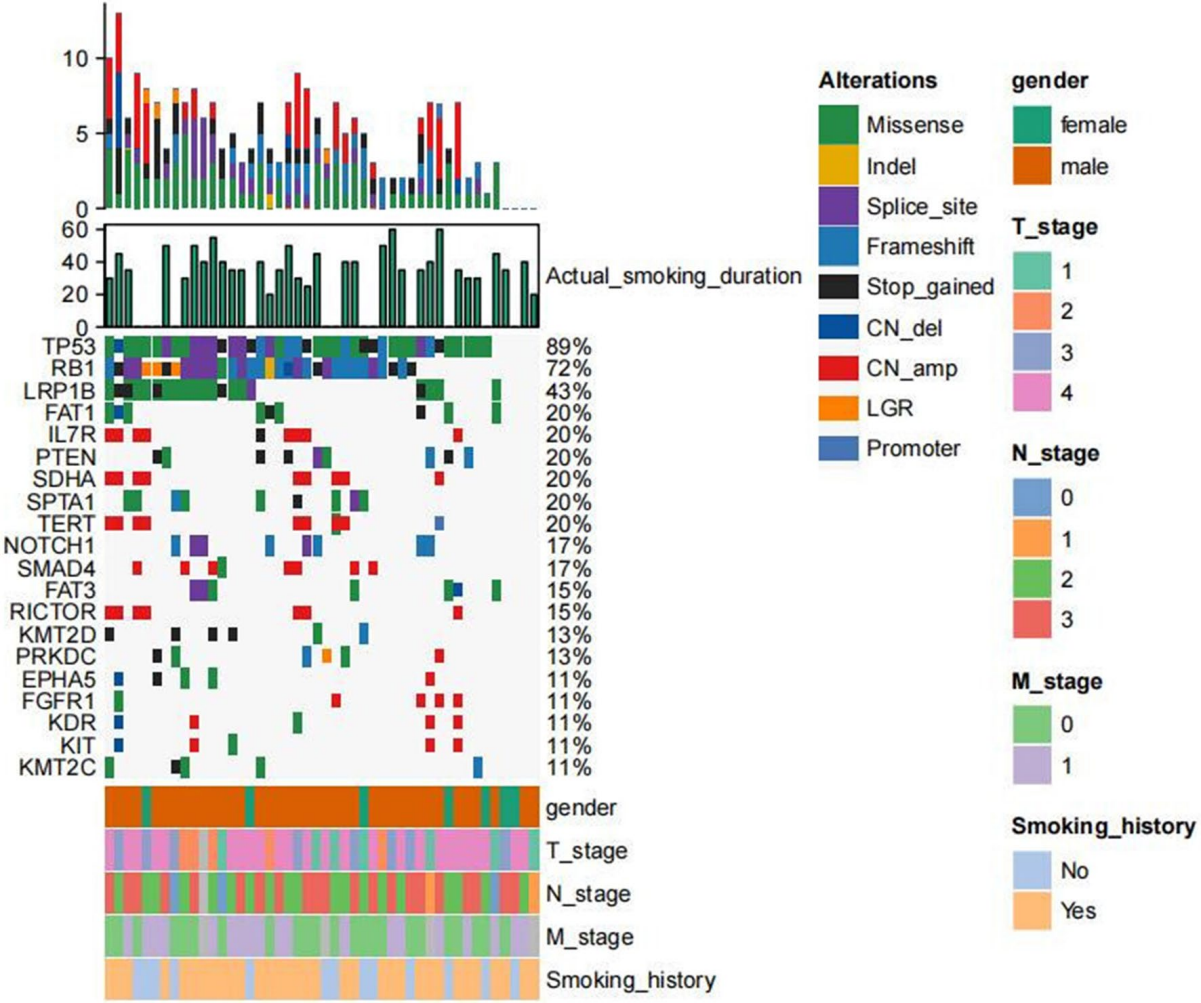
Genomic profiling of 46 patients without EGFR mutations in group B, each column was the relevant tumor samples of the 11 patients, the mutations detected for a given gene were exhibited on each row respectively, and the proportions of them was shown on the right axis. Green indicated missense, yellow indicated inframe insertion or deletion, purple indicated splice site, light blue indicated frameshift, black indicated stop gain, dark blue indicated copy number deletion, red indicated copy number amplification, orange indicated large genomic rearrangement, blue indicated promoter

Results of the molecular analysis are shown in Figs. 2 and 3. RB transcriptional corepressor 1(RB1) and tumor protein P53 (TP53) mutations were universally found in both groups, proportion of mutations of both TP53 and RB1 was 73% and 72% respectively. The mutations of LRP1B, FAT1, IL7R, PTEN, SDHA, SPTA1, TERT, NOTCH1, SMAD4, FAT3, RICTOR, KMT2D, PRKDC, EPHA5, FGFR1, KDR, KIT, KMT2C genes scattered distribution in the control group, while the mutations of ALK, BRCA2, CDKN2A, CREBBP, FAT1, FAT2, JAK2, KDR, KMT2D, LRP1B, MYC, NFKBIA, NKX2-1, NOTCH1, PIK3CA, PTEN, SF3B1 genes scattered distribution in the group A.

### Efficacy of first-line treatment in patients

**Fig. 4:**
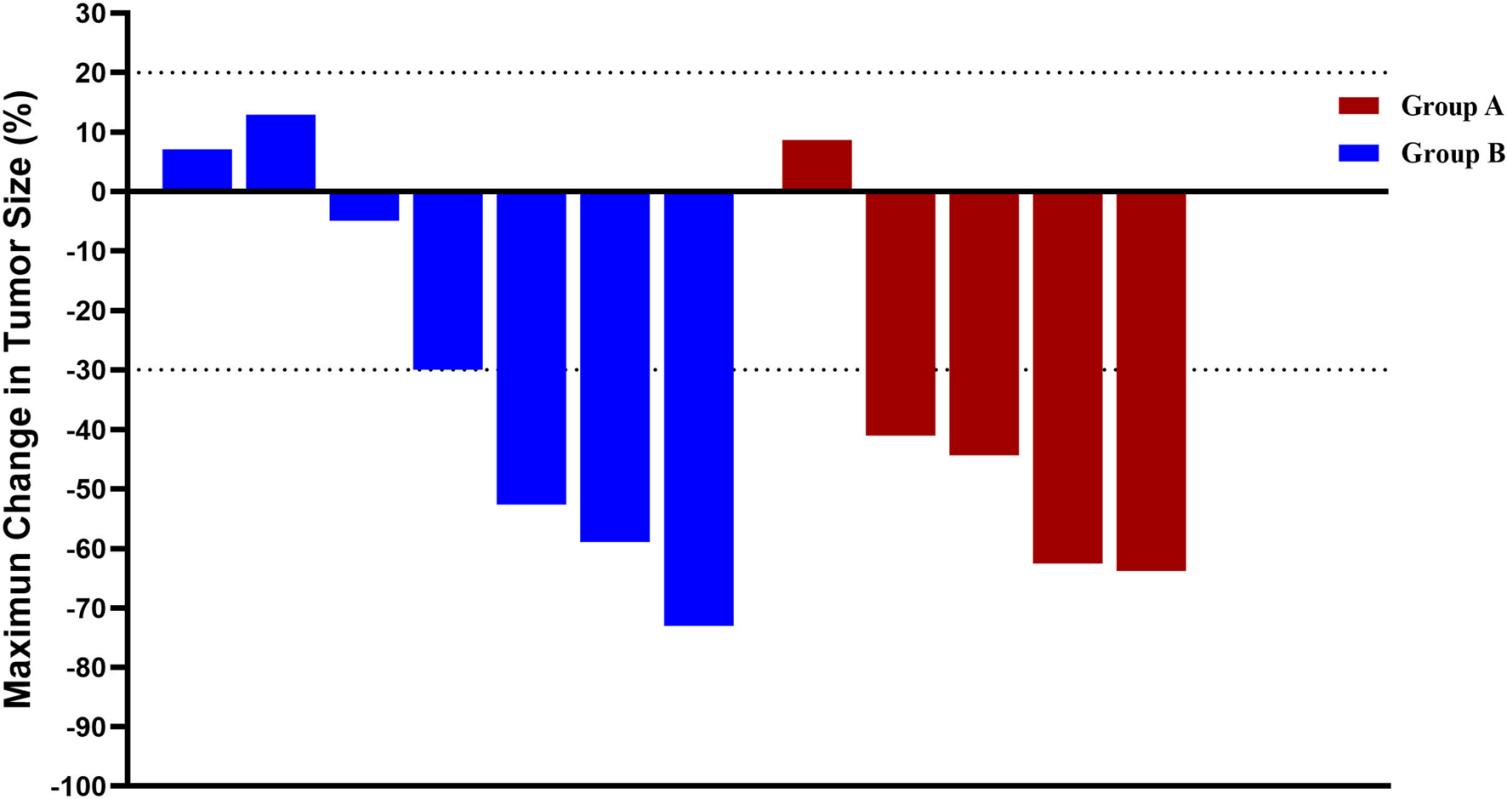
According to RECIST 1.1, maximum percent change from baseline in the sum of the diameter of the longest target lesion in patients with the measurable disease at baseline

**Fig. 5:**
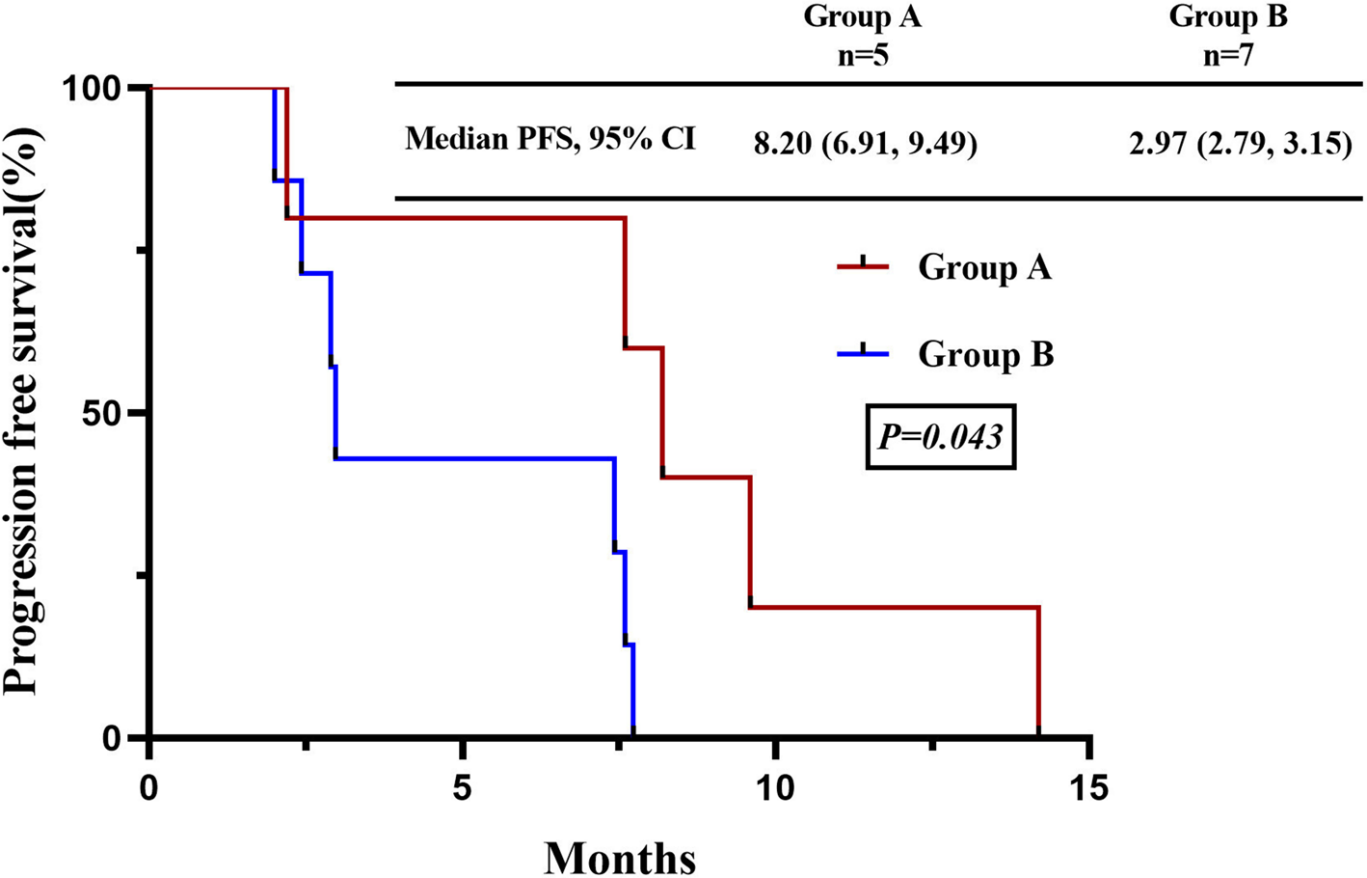
Kaplan–Meier curve for PFS

**Fig. 6:**
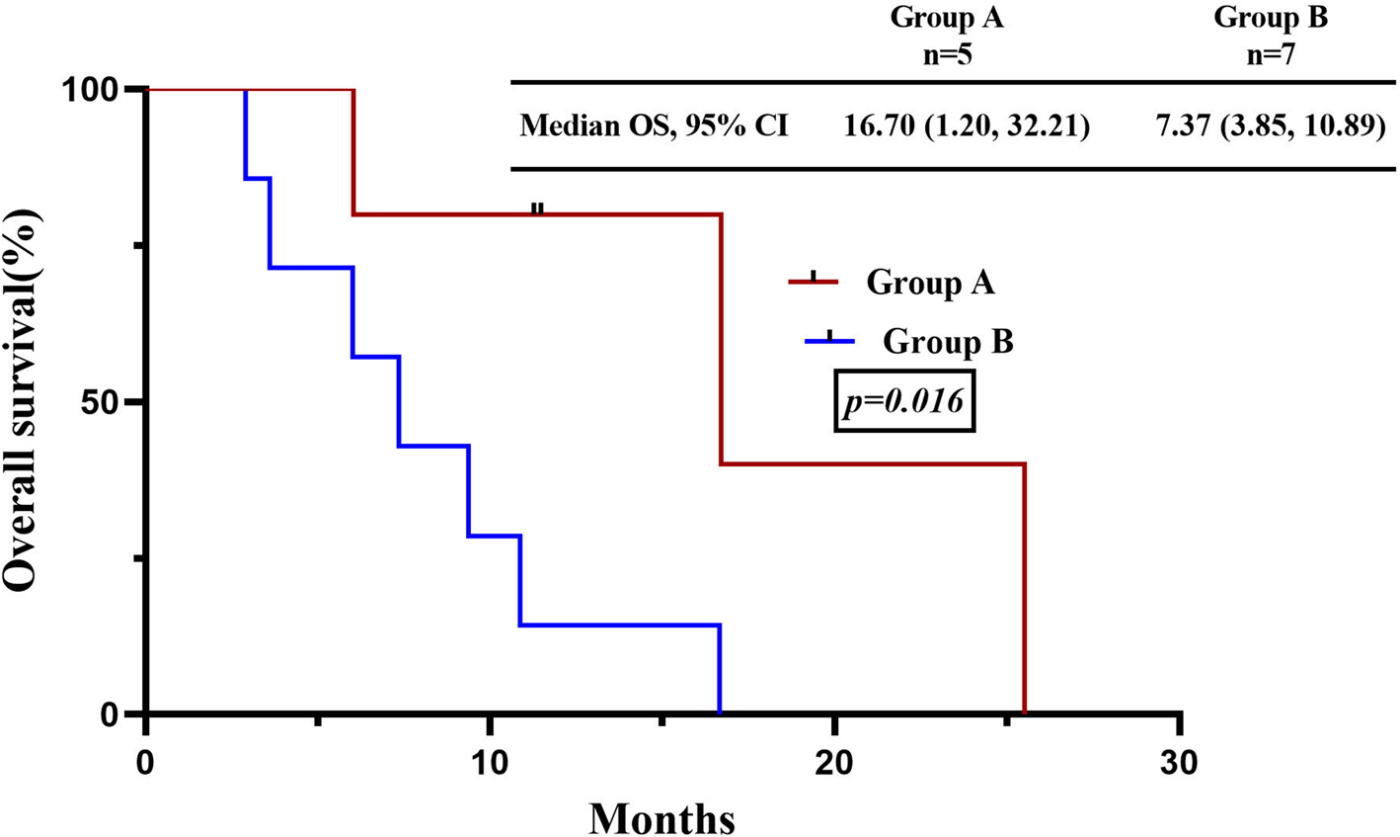
Kaplan–Meier curve for OS

The DCR was 100% in both groups, the ORR was 80% (95% CI 0.13, 0.99) in group A, and 57.1% (95% CI 0.013, 0.987) (Fig. 4). At the time of survival analysis, the median progression-free survival of group A (data eligible for 5 patients, only extensive stage included) in first-line treatment with TKIs plus chemotherapy was 8.20 months (95% confidence interval (CI) 6.91–9.49 months), while 2.98 months (95% confidence interval 2.79–3.15) in group B (data eligible for 7 patients, only EP as the first line therapy and extensive stage have been included), *P* = 0.043. The median overall survival were 16.70 months (95% CI 1.19–32.21) in group A, while 7.37 months (95% CI 3.85–10.89) in group B, *P* = 0.016 Figs. 5, 6.

## Discussion

At present, the reports of EGFR mutation-positive SCLC are still controversial, while histological transformation from non-small cell lung cancer(NSCLC) to small cell lung cancer (SCLC) has been proven to be one of the mechanisms of resistance on EGFR TKIs, occurs in 3 ~ 14% of cases [22–24], and re-biopsy sample showed original EGFR deletion mutation in SCLC components. In addition, combined SCLC, especially combined with adenocarcinoma component would complicate such cases with EGFR mutations. Although limited by the quantity and quality of biopsy specimens, previous studies suggested that EGFR mutations were detected in both adenocarcinoma and small cell lung cancer components in three combined SCLCs [10]. Sutherland et al. had found that knockout of RB1 and TP53 genes in type II alveolar epithelial cells would lead to SCLC, also type II alveolar epithelial cells have the potential to transform into SCLC and lung adenocarcinoma. EGFR is highly expressed in high-differentiated alveolar type II cells, which can be the origin for adenocarcinoma cells carrying EGFR gene mutations [25]. Tatematsu et al. proposed the idea that adenocarcinoma existed prior to the development of SCLC for the EGFR mutant SCLC [10]. Total mentioned above, it suggested that EGFR mutant SCLCs may have developed from preexisting adenocarcinoma with EGFR mutations, which was consistent with proposing differentiated pluripotent cancer stem cells as the putative origin of SCLC [10, 25]. In previous studies, combined SCLC (combined with adenocarcinoma component) were predominated in SCLC which harbored EGFR mutations [9–11], while EGFR mutations were more common in pure SCLC in the present study. It is worth noting, however, that the limitations of biopsy samples would contribute to diagnostic bias.

Clinically, the distributions of sex, smoking history, disease stage, tumor maximum diameter were significantly different. It tended to be female and non-smoker common in the current research, which highly match with the former studies. The immunohistochemical features and molecular analysis of the patients with the mutation showed a trend similar to those of patients without the mutation. In our present study, the proportion of EGFR mutations in SCLC biopsy samples is higher than in the previous studies [7–10]. Among the EGFR mutations group, EGFR 21 (L858R) mutation was found in 4 patients, EGFR 19 (del) mutation in 4, EGFR 18 (G719S) mutation in 1, EGFR 13 (E519D) in 1, EGFR 25(D1009Y) in 1. Among these patients, one harbored EGFR double mutation, and two patients with EGFR mutation and ALK mutation (patient 6 and 11). There were 6 patients who tested samples from initial biopsy and plasma, 1 from pleural effusion and plasma, 2 from plasma and 2 from initial biopsy, all tested samples had proven the existence of EGFR mutations (Table 3).

**Table 3:** Summary of 11 patients with EGFR mutations

Patient No	Sex	Age (years)	Smoke history	EGFR mutations	Tested samples	Histology type	PD-L1 expression	Stage	First-line therapy	Response to FL	OS (m)
1	F	69	No	exon 21 P.L858R	Initial biopsy + plasma	Pure	10% (22C3)	IVA	EP	PR	16.83^※※^
2	F	71	No	exon 21 P.L858R	Initial biopsy + plasma	Pure	10% (22C3)	IVB	EP + TKI	PR	11.47^※^
3	M	77	No	exon 18 G719S	Initial biopsy + plasma	Pure	10% (22C3)	IVB	EP + TKI	PR	16.70^※※^
4	M	48	Yes	exon 19 p.L747_A750de linsP	Pleural effusion + plasma	Pure	1% (22C3)	IVB	EP + TKI	PD	6.03
5	M	46	Yes	exon 19 p.Ala750_l759delinsPT	Initial biopsy + plasma	Pure	0 (22C3)	IVA	EP + TKI	PR	11.27^※^
6	F	53	No	exon 13 E519D	Initial biopsy	Pure	0 (22C3)	IVB	EP + PD-1	PR	21.03^※※^
7	F	62	No	exon 19 p.L747_A75delinsP	Initial biopsy	Pure	NA	IVA	EP + TKI	PR	25.50
8	F	63	No	exon 25 D1009Y	Initial biopsy + plasma	Pure	NA	IVA	EP	PR	16.50^※※^
9	F	67	No	exon 21 P.L858R	Initial biopsy + plasma	Pure	NA	IIIA	EP	PR	6.60^※^
10	M	33	Yes	exon 19 p.Glu746_Ala750del	Plasma	Combined	NA	IVA	EP	PR	21.67
11	M	45	Yes	exon 21 P.L858R	Plasma	Pure	NA	IVB	TKI	NA	8.03

*F* female, *M* male, *EGFR* epidermal growth factor receptor gene, *Del19* exon 19 deletions, *PD-L1* programmed death-ligand 1, *EP*, etoposide plus platinum, *TKI* tyrosine kinase inhibitor, *FL* first-line therapy, *PR* partial response, *PD* progressed disease, *NA* not available

“^※^” Indicates that patients currently alive

“^※※^” Indicates that patients received immune checkpoint inhibitors, regardless of the treatment lines

Regarding molecular analysis, RB1 and TP53 mutations were universally found in both groups, the proportion of mutations of both TP53 and RB1 was 73% and 72% respectively. The mutations of LRP1B, FAT1, PTEN, NOTCH1, KMT2D, KDR, genes scattered distribution in the two groups differently. The mutations of ALK, BRCA2 CDKN2A, CREBBP, FAT2, JAK2, MYC, NFKBIA, NKX2-1, PIK3CA, SF3B1 genes scattered distribution in the group A, while IL7R, SDHA, SPTA1, TERT, SMAD4, FAT3, RICTOR, PRKDC, EPHA5, FGFR1, KIT, KMT2C presented in group B.

Currently, there is no unified standard for the treatment of EGFR mutated SCLC and the efficacy of EGFR-TKIs in EGFR-mutated SCLCs remained unknown, only isolated cases reported that gefitinib is effective in such patients. We summarized 5 cases had administrated EP combined with TKIs in first-line therapy in group A. Therefore, in the evaluation of efficacy, only first-line EP combined with TKI in group A and only EP as the first-line strategy, extensive stage, without radiotherapy in group B were enrolled. Compared to the EGFR-wild group, both PFS and OS were better in the EGFR mutations group, whether it is due to EGFR mutations is still unknown. To our best knowledge, this is the first research to evaluate the treatment efficacy of EGFR mutant SCLC comprehensively.

EGFR-mutated patients in our study had longer OS than EGFR wild-type even though they were not treated with EGFR-TKI, suggesting a potential favorable prognostic role of EGFR mutations in SCLC. The therapeutic strategies for the intractable SCLC had unchanged for several decades. AS a giant breakthrough in oncology in recent years, immune checkpoint inhibitors have become one of the most potential treatments for patients with several solid cancers. Based on IMPower133 study [26], USA Food and Drug Administration (FDA) approved the combination of Atezolizumab with standard chemotherapy as the first-line regimen in extensive-stage SCLC in March 2019 and immunotherapy showed a promising landscape in the prognosis of SCLC. In addition, patient 1, 3, 6 and 8 were administrated for immune checkpoint inhibitors, regardless of the lines of treatment, have better OS (the median OS was 16.77 months), which sparked our interest in exploring the immune microenvironment and gene expression in this type of tumor samples. However, in the current study, we extracted RNA from the biopsy samples failed due to the limited samples, which cannot continue the next step on RNA-seq analysis.

In conclusion, EGFR-mutated SCLC patients tended to be female and non-smoker, they shared similar pathology and molecular features to the conventional SCLC and experienced a prolonged OS suggesting a possible positive prognostic effect. Further research is warranted to explore the potential association of the tumor microenvironment for such SCLC with EGFR mutations and outcrop suitable treatment strategies.

## Data Availability

Data presented in this study are included in the article/supplementary material. Available at (https://www.biosino.org/node/project/detail/OEP003617).

## Abbreviations

ALK: Anaplastic lymphoma kinase
BRCA2: BRCA2 DNA repair associated
CK: Cytokeratin
CgA: Chromogranin A
CD56: Nerve cell-associated adhesion molecule
CDKN2A: Cyclin dependent kinase Inhibitor 2A
CREBBP: CREB binding protein
CI: Confidence interval
EGFR: Epidermal growth factor receptor
EP: Etoposide plus platinum
EPHA5: EPH receptor A5
FGFR1: Fibroblast growth factor receptor 1
FAT1: FAT atypical cadherin 1
FAT3: FAT atypical cadherin 3
IHC: Immunohistochemistry
IL7R: Interleukin 7 receptor
JAK2: Janus kinase 2
KDR: Kinase insert domain receptor
KIT: KIT proto-oncogene
KMT2C: Lysine methyltransferase 2C
KMT2D: Lysine methyltransferase 2D
LRP1B: Low-density lipoprotein receptor-related protein 1B
MYC: MYC proto-oncogene, BHLH transcription factor, nuclear factor-kappa B
NFKBIA: NFKB Inhibitor Alpha
NKX2-1: NK2, homeobox 1
NSCLC: Non-small lung cancer
NOTCH1: Notch receptor 1
NGS: Next-generation sequencing technology
OS: Median overall survival
PRKDC: Protein kinase, DNA-activated, catalytic subunit
PTEN/MMAC1: Mutated in multiple advanced cancers 1
PFS: Median progression-free survival
PIK3CA: Phosphatidylinositol-4,5-bisphosphate 3-kinase catalytic subunit alpha
RECIST: Response evaluation criteria in solid tumors
RB1: RB transcriptional corepressor 1
RICTOR: RPTOR independent companion of MTOR complex 2
Syn: Synaptophysin
SMAD4: SMAD family member 4
SHDA: Succinate dehydrogenase complex flavoprotein subunit A
SPTA1: Spectrin alpha, erythrocytic 1
SF3B1: Splicing factor 3b subunit 1
SCLC: Small cell lung cancer
TERT: Telomerase reverse transcriptase
TTF1: Thyroid transcription factor-1
TKIs: Tyrosine kinase inhibitors
TP53: Tumor protein P53
WHO: World health organization
